# Evaluation des résultats après traitement des lésions intra épithéliales du col utérin par la cryothérapie: étude préliminaire au Centre Hospitalier Universitaire de Yaoundé: A propos de 21 cas

**DOI:** 10.11604/pamj.2015.20.225.4618

**Published:** 2015-03-12

**Authors:** Claude Cyrille Noa Ndoua, Pierre Marie Tebeu, Jean Dupont Kemfang, Jean Marie Kasia

**Affiliations:** 1 Faculté de Médecine et Sciences Biomédicales de l'Université de Yaoundé I, Cameroun; 2 Centre Hospitalier de Recherche et d'Application de la Chirurgie Endoscopique et de la Reproduction Humaine, Cameroun; 3 Centre Hospitalier et Universitaire de Yaoundé, Cameroun; 4 Hôpital Général de Yaoundé, Cameroun

**Keywords:** Cryothérapie, lésions intra épithéliales, colposcopie, Cryotherapy, intraepithelial lesions, colposcopy

## Abstract

Nous rapportons les résultats d'une série de 21 cas de prise en charge par cryothérapie de lésions intra-épithéliales cervicales au Centre Hospitalier et Universitaire (CHU) de Yaoundé. Notre objectif principal était d’évaluer les résultats préliminaires de la prise en charge des lésions précancéreuses éligibles pour la cryothérapie. Il s'agissait d'une étude transversale descriptive qui s'est étalée sur 24 mois. Etaient inclus dans l’étude toutes les femmes traitées par cryothérapie. Nous avons exclu les patientes traitées par une autre méthode, les patientes perdues de vue et les dossiers incomplets. Le statut cervical a été déterminé à 6 semaines, 6 mois et 12 mois. Les complications précoces et tardives ont également été répertoriées. Au total 95.2% des lésions étaient cicatrisées à 6 semaines. A 6 mois, toutes les lésions avaient disparu et au 12^ème^ mois, la guérison était effective chez 95.2% des patientes. Les saignements et l'hydrorrhée étaient les principales complications tardives avec des fréquences respectives de 66.7% et 95.2%. Aucun cas de sténose cervicale n'a été répertorié. La cryothérapie peut être utilisée comme méthode de traitement pour des lésions précancéreuses du col.

## Introduction

Le cancer du col est la deuxième cause de mortalité par cancer chez la femme dans les pays en voie de développement (11.4%) [1]. Il s'agit du cinquième cancer dans population générale après le sein, la prostate, les poumons et le colon. Selon l'OMS l'incidence annuelle était 530 000 nouveaux cas dans le monde en 2008 dont plus de 90% dans les pays en voie de développement [1]. L’âge moyen de survenue est de 52,5 ans. Au Cameroun, c'est le deuxième cancer gynécologique (23.4%) après le sein et l'incidence annuelle est 24/100000 [1]. L´infection persistante à HPV à haut risque oncogène est considérée comme la cause du cancer du col utérin [2]. L'infection génitale par un HPV est une des infections sexuellement transmissibles (IST) les plus répandues chez les femmes jeunes sexuellement actives. Le nombre de nouvelles infections génitales par un HPV dans le monde est estimé à 30 millions par an. Il est estimé que 50 à 75% des femmes de 15 à 44 ans sont ou ont été exposées aux HPV [3]. L´histoire naturelle du cancer du col de l'utérus comporte plusieurs lésions histologiques précancéreuses (les néoplasies cervicales intra-épithéliales ou CIN), faisant suite à la persistance de l'infection génitale par un HPV à haut risque oncogène, dont certaines sont des stades facultatifs (CIN 1 et CIN 2) et d´autres des étapes nécessaires (CIN 3) à l'apparition d'un cancer invasif [4]. La prévalence des lésions précancéreuses dans la population camerounaise 7-8% [5, 6]. Environ 22.16% de ces lésions confirmées sont de haut grade et imposent un traitement (Tebeu). Au CHU de Yaoundé un programme de dépistage a été mis en place permettant un diagnostic et une prise en charge précoce des lésions précancéreuses. La prise en charge fait appel à trois attitudes thérapeutiques: l'abstention et surveillance; la résection qui peut être faite au bistouri froid ou à l'anse diathermique; la destruction avec des méthodes telles que l’électrocoagulation, la vaporisation au laser et la cryothérapie, objet de notre étude. La cryothérapie a été introduite au CHU de Yaoundé en 2011, permettant à cette formation hospitalière d'offrir aux patientes l'essentiel de l'arsenal thérapeutique indiqué dans la prise en charge des lésions précancéreuses. Notre travail consiste donc à évaluer les résultats préliminaires de cette attitude thérapeutique depuis son introduction au CHU. Le CHU de Yaoundé s'est doté du matériel de cryothérapie en 2011 et certaines patientes présentant des lésions précancéreuses ont été prises en charge depuis l'introduction de cette nouvelle technique. Aucune étude jusqu´à présent n'a été réalisée pour évaluer les résultats en matière de guérison, récidive ou complications après cryothérapie d'où la nécessité pour nous de mener cette étude préliminaire. La question de recherche était quel serait le statut cervical des femmes traitées par cryothérapie? Nous émettons l'hypothèse selon laquelle les femmes traitées par cryothérapie ont une disparition totale des lésions au bout de 12 mois. l'objectif principal était d’évaluer les résultats préliminaires de la prise en charge des lésions précancéreuses par la cryothérapie au CHU de Yaoundé. et les objectifs spécifiques, déterminer le profil sociodémographique des patientes traitées par la cryothérapie; Identifier les caractéristiques des lésions cervicales traitées par cryothérapie; de déterminer le pourcentage des lésions non cicatrisées; après cryothérapie, d'identifier les complications de la cryothérapie; de donner le pourcentage des lésions disparues après 6 mois; et de déterminer le pourcentage de guérison après 12 mois.

## Méthodes

### Lieu d’étude

Le Service de gynécologie/obstétrique du Centre Hospitalier Universitaire de Yaoundé ou un programme de dépistage et de prise en charge par cryothérapie a été mis en place depuis 2011. Le CHU de Yaoundé dispose d'une unité de prise des lésions précancéreuses du col logée au sein du service de Gynécologie. Celle-ci est coordonnée par le chef de service. L’équipe opérationnelle comprend un gynécologue obstétricien qu'assistent un médecin généraliste et une infirmière.

### Type d’étude

Il s'agissait d'une étude descriptive et transversale.

### Durée de l’étude

Elle a porté sur 24 mois allant de Janvier 2011 à décembre 2012.

### Population d’étude

Population cible: il s'agissait de patientes reçues au CHU pour lesion précancéreuse.

Critères d'inclusions: toutes les femmes présentant une lésion précancéreuse traitée par cryothérapie.

Critères d'exclusion: les femmes traitées par une autre méthode; les patientes perdues de vue; les dossiers incomplets.

**Echantillonnage**: Le recrutement était consécutif et la taille de l’échantillon a été déterminée a la fin du recrutement.

### Procédure

#### Variables

Les variables suivantes ont été déterminées: Les caractéristiques sociodémographiques des patientes, La taille des lésions, La cytologie des lésions et le type histologique des lésions, L’état cervical à 6 semaines, 6 mois et 12 mois, Les complications précoces et tardives. Il s'est agi de l'analyse des dossiers des malades colligés et du suivi des patientes prise en charge par cryothérapie au CHU. Apres confirmation de la lésion et localisation de celle-ci par le test de VIA/VILI, un traitement par cryothérapie dont les étapes sont listées ci-contre a été effectué. Installation de la patiente en position gynécologique et faire un examen au spéculum. Nettoyage du col avec du sérum physiologique. Application de l'acide acétique à 5% pour bien délimiter l'anomalie sous colposcopie. Application du Lugol. Nettoyage de la surface de la sonde cryogénique avec du sérum physiologique. Mise en place de l'embout de la sonde cryogénique dans le canal cervical et s'assurer que celle-ci couvre correctement la lésion. Faire 2 cycles de congélation-décongélation: congélation de 3 minutes; Suivie d'une décongélation de 5 minutes; Puis une nouvelle congélation de 3 minutes; Enfin une décongélation. Retirer la sonde cryogénique, retirer le spéculum, hémostase si le col saigne. Ensuite nous avons procédé à une évaluation de la cicatrisation à 6 semaines. Si la cicatrisation était satisfaisante, la patiente était reçue au 6e et 12e mois pour bénéficier d'un examen coelioscopique qui permettait d’évaluer l'absence ou la persistance de la lésion. Toutefois, les lésions non cicatrisées à la 6e semaine étaient réévaluées au 3e mois. De même, les lésions persistantes étaient retraitées. Les complications immédiates et tardives ont été répertoriées.

#### Matériel utilisé

Sur le terrain: fiches techniques individuelles; Dossiers médicaux; Logistique (crayons, stylo à bille, gommes…); Matériel de cryothérapie

**Analyses statistiques**: Les données ont été analysées par ordinateur grâce au logiciel épi info visant à calculer les moyennes, les pourcentages, l’écart type,…qui seront représentés sous forme d'histogrammes et de tableaux.

## Résultats

### Caractéristiques sociodémographiques des patientes

L’âge moyen de notre échantillon était de 39.9 ± 5.4. La tranche d’âge la plus représentée était celle des moins de 45 ans avec 15 cas soient 71.4%. Deux patientes soient 9.5% avaient plus de 55 ans et 4 patientes soient 19% avaient plus de 45 ans. Le dépistage concernait les femmes âgées de 25 à 65 ans raison pour laquelle dans notre échantillon aucune femme n'avait plus de 65 ans. Concernant le niveau d'instruction 42.8% des patientes avaient fait des études supérieures. Le taux restant était équitablement reparti entre les femmes des niveaux primaire et secondaire. Les femmes d'un niveau d'instruction plus élevé étaient plus conscientes des bénéfices du dépistage dans la prise en charge du cancer du col. La majorité des patientes soient 85.7% étaient mariées. Elles sont pourtant moins à risque les célibataires. La plupart des femmes mariées affirmaient avoir été encouragées par leurs époux à faire le dépistage (Tableau 1).

**Tableau 1:** Caractéristiques sociodémographiques des patientes

Paramètres		Effectifs/21	Pourcentage
**Age**	25-35	5	23.8
	36-45	10	47.6
	46-55	4	19
	56-65	2	9.6
**Niveau d'instruction**	Primaire	6	28.6
	Secondaire	6	28.6
	Supérieur	9	42.8
**Statut matrimonial**	Mariée	18	85.7
	Veuve	1	9.5
	Célibataire	2	4.8

### Circonstances de découverte de la lésion cervicale

La campagne de dépistage a permis de recruter 18 cas soient 85.7%. Les 3 autres cas ont été dépistés après la survenue de symptômes évocateurs. Il s'agissait de metrorragies. Le coût dépistage du cancer du col dans notre milieu limite la pratique de celui-ci. La campagne de dépistage attire d'avantage les patientes car elles sont assurées d'une prise en charge gratuite en cas de lésions (Tableau 2).

**Tableau 2:** Répartition de la population suivant les circonstances de découverte de la lésion cervicale

Circonstances	Effectifs	Pourcentage
Lors du dépistage	18	85.7
Lésion symptomatique	3	14.3
Total	21	100

### Caractéristiques des lésions

Nous avons diagnostiqué 14 cas de LSIL soient 66.7% et 5 cas de HSIL soient 23.8% parmi lesquels 2 étaient symptomatiques. Cependant 2 patientes n'ont pas fait de frottis elles se sont présentées d'emblée avec des résultats de biopsie pathologiques. Les tests de VIA et VILI étaient positifs chez toutes les patientes devant. A l'histologie, 66.7% des lésions étaient des CIN II. Nous avons diagnostiqué 5 cas de CIN I et 2 cas de CIN III. Nous avons enregistré 6 cas de condylome et 1 cas de cervicite. Le cas de cervicite retrouvé a été initialement traité avant la cryothérapie. La classification topographique des lésions précancéreuses distingue la lésion de type I entièrement exocervicale, la lésion de type II dont le bord interne affleure l'os externe du col et la lésion de type III se prolongeant dans l'endocol. La cryothérapie est contre-indiquée pour les lésions de type III remontant à plus de 3 mm dans le canal cervical. Ainsi, 95.2% des patientes que nous avons prises en charge avaient des lésions de type I ou type II. La majorité soit 95.2% des lésions avaient un diamètre entre 1 et 2 cm. Seule une lésion mesurait moins de 1 cm. La taille de la lésion doit etre inferieure au diamètre de la plus grande cryode qui mesure 2.5 cm (Tableau 3).

**Tableau 3:** Répartition selon les caractéristiques des lésions

Caractéristiques		Effectifs/21	Pourcentage
Cytologie	LSIL	15	71.4
	HSIL	6	28.6
Colposcopie	VIA Positif	21	100
	VILI Positif	21	100
Biopsie	CIN I	5	23.8
	CINII	14	66.7
	CIN III	2	9.5
Lésions associées	Cervicite	1	4.8
	Condylome	6	28.6
Localisation de la lésion	Type 1	4	19
	Type 2	16	76.2
	Type 3	1	4.8
Taille de la lésion en cm	< 1 cm	1	4.8
	Entre 1 et 2 cm	20	95.2

### Répartition de la population suivant le délai entre le diagnostic et la prise en charge

Dix- huit patientes soient 85.7% ont été prise en charge dans les 3 premiers mois suivant le dépistage. La campagne de dépistage qui se veut gratuite impose la disponibilité d'un certain matériel qui n'est pas toujours présent au moment du diagnostic. De ce fait, les patientes sont reconvoquées par la suite (Tableau 4).

**Tableau 4:** Répartition de la population suivant le délai entre le diagnostic et la prise en charge

Délai	Effectifs	Pourcentage
Immédiat	6	28,6
dans les 3 mois	12	57,1
3-6 mois	2	9,5
> 6 mois	1	4,8
Total	21	100

### Répartition de la population suivant les Complications

Les complications immédiates ont été répertoriées dans les 24 heures. Au total 95.2% des patientes ont présenté un saignement immédiat pris en charge par simple compression. Nous avons enregistré 13 cas de douleurs immédiates post thérapeutiques et 5 cas de crampes. L'hydrorrhée était la principale complication tardive enregistrée avec 95.2% des cas. Par ailleurs nous avons enregistré 14 cas de saignement soient 66.7%. Il s'agissait de saignement persistant ou de saignement secondaire après 24 heures. Nous n'avons enregistré aucun cas de sténose cervicale ni de fièvre (Tableau 5).

**Tableau 5:** Répartition selon les complications

Complications		Effectifs/21	Pourcentage
Immédiates	Crampes	5	23.8
	Saignement	20	95.2
	Douleurs	13	61.9
Tardives	Saignement	14	66.7
	Douleurs	7	33.3
	Leucorrhées	7	33.3
	Hydrorrhées	20	95.2
	Sténose cervicale	0	0

**Evaluation de la cicatrisation apres 6 semaines** Une seule lésion n'a pas présenté une cicatrisation satisfaisante apres 6 semaines tandis que les 20 autres soient 95.2% étaient complètement cicatrisées (Tableau 6).

**Tableau 6:** Répartition suivant l’état de la cicatrice après 6 semaines

Cicatrisation complète	Nombre	pourcentage
Oui	20	95.2
Non	1	4.8
Total	21	100

### Contrôle VIA/VILI à distance

A 3 mois, une patiente a bénéficié d'un test de VIA/VILI pour mauvaise cicatrisation à la 6e semaine. Dans son cas, les tests VIA/VILI étaient négatifs au 3e mois. Au total, quinze patientes ont été revues à 6 mois et 12 mois. Une seule a été revue au 18e mois car elle avait un test VIA/VILI positif au 12e mois. Elle a immédiatement bénéficié d'un 2e traitement (Tableau 7).

**Tableau 7:** Répartition selon les résultats du VIA/VILI à distance

	A 3 mois	A 6 mois	A 12 mois	A 18 mois
Nombre de patientes vues	1	21	21	1
Absence de lésion	1	21	20	1
Persistance	0	0	1	0

## Discussion

### Caractéristiques socio démographiques des patientes

Nos cas ont été recrutés à partir d'un bassin sélectif sur une courte période de 2 ans. Il s'agit dune étude préliminaire depuis l'introduction de la cryothérapie au CHU qui fait désormais partie de l'arsenal thérapeutique de prise en charge des lésions précancéreuses. Au total 27 patientes ont été prise charge pour lésion précancéreuse parmi lesquelles, 21 éligibles à la cryothérapie soient 77.7%. Cinq patiente ont bénéficié d'une conisation tandis qu'une patiente non éligible à la cryothérapie était perdue de vue. Quinze patientes ont été suivies jusqu´à 12 mois et 6 patientes sont en attentes de la première évaluation du 6e mois. Notre travail a consisté à définir les caractéristiques socio démographiques de ces patientes et à évaluer le résultat précoce et tardif après traitement. La moyenne d’âge de nos patientes est de 39.9 ± 5.4. La proportion des femmes de moins de 45 ans est de 71.4%. Même s'il s'agit d'une sélection exclusive des femmes éligibles à la cryothérapie, cet âge moyen se rapproche de 40 ans rapporté dans la population générale des femmes présentant une lésion précancéreuses par Tebeu et all en 2005 [6]. Dans la série de Poomtavom [7], la moyenne d’âge des patientes éligibles pour la cryothérapie était de 36.2. Ainsi, l’âge moyen varie selon les études et serait fonction de l’âge moyen de survenue de cancer invasif au sein d'une population donnée. Généralement, les lésions précancéreuses précédentes de 10 à 15 ans les cancers invasifs [8–10]. Notre résultat obéit à cette logique car l’âge moyen de survenue des cancers invasifs au Cameroun est d'environ 50 ans [11]. La majorité des patientes de notre série sont mariées. Tebeu a trouvé un résultat similaire dans sa série [6]. Il n'est point question d'une population à risque supplémentaire car cette forte proportion trouve son explication par le fait que ces dernières sont davantage encouragées par leurs époux à faire le dépistage comme elles nous l'ont affirmé. Cette observation est similaire à celle rapportée par Bertolotto [12] Le taux élevé des patientes ayant fait des études supérieures dans notre série (42.8%) est plus attribué à l'effet positif du niveau intellectuel sur la participation aux campagnes de dépistage [12].

### Circonstances de découverte de la lésion précancéreuse

Les lésions précancéreuses sont généralement asymptomatiques et ne sont découvertes pour la plupart que lors d'un dépistage organisé ou personnel. C'est pourquoi 85.7% de nos cas sont issus d'une campagne de dépistage. Certaines patientes peuvent présenter une symptomatologie à type de leucorrhées, métrorragie. Elles ne sont pas nombreuses. Dans notre série 3 cas ont été diagnostiqués à la faveur d'une symptomatologie évocatrice.

### Diagnostic et caractéristiques de la lésion précancéreuse

Quelques soit le mode de découverte, toutes nos patientes ont bénéficié d'un frottis cervical, suivi d'une colposcopique si le frottis était anormal puis dune biopsie en fonction du résultat de la colposcopie. Sur le plan histologique, les LSIL représentaient 71.4% et les HSIL 28.6%. Notre résultat se rapproche de celui rapporté par Tebeu et all sur les lésions précancéreuses [6]. Les tests de VIA/VILI étaient positifs pour tous les cas. A la biopsie, la répartition était de 23.8% CIN I, 66.7% CIN II et 9.5% CIN III. Au total 11 cas de LSIL (73.3%) sur les 15 se sont avérés être soit des CIN II ou des CIN III. Ce qui met en lumière l'importance de la biopsie dans le diagnostic définitif des néoplasies cervicaux. En effet, comme le rapporte Hunter [13], il n'y à pas de correspondance cyto-histologique franche dans le diagnostic des lésions précancéreuses car plusieurs lésions initialement LSIL se trouvent être soit des CIN II soit CIN III. S'agissant de la taille de la lésion, seules les lésions dont le diamètre est inférieure à celui de la plus grande cryode sont éligibles à la cryothérapie. La plus grande cryode que nous avons utilisée avait un diamètre de 2.5 cm raison pour laquelle toutes les lésions traitées dans notre série ont moins de 2 cm. De plus, la cryothérapie est plus efficace sur des lésions de petite taille [14]. Cette méthode a généralement un taux de guérison plus faible pour les lésions plus larges que la sonde utilisée pour la cryothérapie ou occupant, en moyenne, plus de 75% de la surface du col utérin [15]. La plupart des lésions que nous avons traitées étaient de localisation extra cervicale soient 95.2%. La seule lésion avec extension endocervicale que nous avons traitée s’étendait sur 2 mm dans le canal cervical. La plupart des auteurs s'accordent sur la nécessité d'une localisation essentiellement exocervicale pour une prise en charge par la cryothérapie [15, 16]. Cette exigence n'est cependant pas absolue car Sellors recommande une prise en charge des tout de même des lésions dont l'extension dans le canal n'excède pas 2 à 3 mm.

### Prise en charge, complications et suivi

Il n'est pas toujours nécessaire de traiter une lésion précancéreuse. Selon sa gravité, deux options sont possibles: surveiller la lésion jusqu’à ce qu'elle guérisse spontanément ou la supprimer. Seulement, le problème de surveillance se pose dans notre milieu raison pour laquelle lors des campagnes de dépistage, les lésions précancéreuses doivent pour la plupart être traitées. De plus, Le dépistage ne sera d'aucune utilité s'il n'est pas suivi de la mise en ‘uvre d'un traitement efficace chez celles qui présentent la maladie [17]. Ainsi, dix-huit patientes représentant 85.7% des cas ont été immédiatement prise en après le diagnostic. Un cas a été pris en charge après un délai de 6 mois. Le risque d’évolution d'une lésion précancéreuse vers une lésion invasive est minime dans un délai de 6 mois car l'histoire naturelle de la maladie montre que celle-ci se fait en 10-15 ans [10]. Deux cycles de cryothérapie ont été utilisés pour chaque patiente. Aucun cas n'a nécessité un troisième cycle de cryothérapie qui est généralement obligatoire lorsque la boule de glace formée ne dépasse pas l'embout de la sonde [17]. Nous avons répertorié deux groupes de complications. Les complications immédiates ou précoces qui surviennent dans les 24 heures et les complications tardives. S'agissant des complications immédiates, 20 patientes soient 95.2% ont présenté des saignements minimes ou modérés. Les autres complications immédiates sont les douleurs dans 61.9% cas et les crampes dans 23.8% des cas. Comme complications tardives nous avons enregistré 20 cas d'hydrorrhée soient 95.2%, 14 cas de saignements tardifs 7 cas de leucorrhées et 7 cas de douleurs. Lewis [17] rapporte des résultats similaires concernant la survenue d'hydrorrhée. La fréquence des saignements reste élevée dans notre série. Elle serait liée à un congélation trop importante si bien que la boule de glace dépasserait les 5 mm de profondeur tel que décrit dans la littérature [18]. Nous n'avons enregistré aucun cas de sténose cervicale. En effet, aucune preuve n'a été faite qu'il existe un lien entre la cryothérapie et la sténose cervicale ou bien que la cryothérapie a un impact à long terme sur la fertilité des femmes ou sur leurs grossesses futures [15].

En ce qui concerne la sûreté, Castro [14] suggère qu'il est très peu probable que des complications majeures, telles que de graves hémorragies et la maladie inflammatoire pelvienne, se produisent à la suite d'une cryothérapie. Ces complications majeures se produisent moins souvent après une cryothérapie qu'après une résection à l'anse ou un traitement par ablation laser. Gage [18] rapporte un cas d'hémorragie sévère chez une patiente ayant reçu un traitement de cryothérapie. La cicatrisation a lieu dans les six premières semaines qui suivent la cryothérapie et la guérison confirmée à 12 mois [17]. La majorité de nos patientes ont cicatrisé dans les 6 semaines. Une seule lésion n’était pas complètement cicatrisée dans ces délais et devait de ce fait bénéficier d'une évaluation précoce à 3 mois contrairement aux autres qui ont été évaluées après 6 mois. A 6 mois l'examen colposcopique était normal chez toutes les patientes tandis que le taux de guérison à 12 mois dans notre série était 95.2% tout type histologique confondu. Le taux de guérison des lésions CIN I était de 100% alors que nous avons enregistré 93.7% de guérison pour les lésions CIN II et CIN III. Persad [19] rapporte un taux de guérison global de 91.6%. Une étude en Afrique du Sud a rapporté des taux de guérison de plus de 90% [20]. Une autre étude en Inde a révélé des taux de guérison similaires, même lorsque des infirmières, plutôt que des médecins, avaient effectué la cryothérapie [21]. La lésion qui a persisté dans notre série était un CIN II entièrement exocervicale. Une série péruvienne a retrouvé des taux de guérison de plus de 90% pour les CIN 1 et 2 et de 70% pour les CIN 3 dans les trois ans suivant le traitement par cryothérapie [19] ce qui pose le problème de la définition même de la récidive. Elle traduit en effet une lésion qui apparais après 12 mois alors que les contrôles subséquents étaient négatifs. Toutes les lésions CIN III n’étaient plus présentes au 12e mois. Nos résultats confirment l'efficacité de la cryothérapie sur les lésions intra épithéliales de haut grade telle que rapportée par plusieurs auteurs [14, 22].

## Conclusion

La cryothérapie est une modalité de traitement efficace qui devrait faire partie de la prise en charge du cancer du col utérin pré-invasif. Nous avons trouvé un taux de cicatrisation à 6 semaines de 95.2% et la guérison était effective chez 93.3% des patientes au 12e mois. Le saignement et l'hydrorrhée étaient les principales complications tardives. Ainsi, la mise en ‘uvre d'un test de dépistage suivi d'une cryothérapie compte l'avantage d'assurer la prise en charge des lésions précancéreuses au niveau des soins primaires, sans devoir recourir à du matériel sophistiqué ou à du personnel médical hautement spécialisé. Cette approche permet également d'assurer que les femmes reçoivent un traitement immédiat et de réduire le risque de perte de vue. Proposer un traitement par cryothérapie aux femmes ayant un résultat de dépistage positif lors de la même visite peut être une approche rationnelle dans certaines régions. Il est important que des unités de cryothérapie efficaces et abordables en nombre suffisant soient rendues accessibles aux programmes de prévention du cancer du col de l´utérus.
